# Health effects of smoke from planned burns: a study protocol

**DOI:** 10.1186/s12889-016-2862-y

**Published:** 2016-02-24

**Authors:** David O’Keeffe, Martine Dennekamp, Lahn Straney, Mahjabeen Mazhar, Tom O’Dwyer, Anjali Haikerwal, Fabienne Reisen, Michael J. Abramson, Fay Johnston

**Affiliations:** Department of Epidemiology and Preventive Medicine, School of Public Health and Preventive Medicine, Monash University, Level 6 The Alfred Centre, 99 Commercial Road, Melbourne, 3004 Australia; CSIRO Oceans and Atmospheric Flagship, Private Bag 1, Aspendale 3195, Victoria, Australia; Menzies Research Institute Tasmania, Medical Science Precinct, University of Tasmania, 17 Liverpool Street, Hobart, 7000 Australia

**Keywords:** Planned burn smoke, Forest fire smoke, Lung inflammation, Endothelial function, Markers of inflammation, Particulate matter (PM)

## Abstract

**Background:**

Large populations are exposed to smoke from bushfires and planned burns. Studies investigating the association between bushfire smoke and health have typically used hospital or ambulance data and been done retrospectively on large populations. The present study is designed to prospectively assess the association between individual level health outcomes and exposure to smoke from planned burns.

**Methods/design:**

A prospective cohort study will be conducted during a planned burn season in three locations in Victoria (Australia) involving 50 adult participants who undergo three rounds of cardiorespiratory medical tests, including measurements for lung inflammation, endothelial function, heart rate variability and markers of inflammation. In addition daily symptoms and twice daily lung function are recorded. Outdoor particulate air pollution is continuously measured during the study period in these locations. The data will be analysed using mixed effect models adjusting for confounders.

**Discussion:**

Planned burns depend on weather conditions and dryness of ‘fuels’ (i.e. forest). It is potentially possible that no favourable conditions occur during the study period. To reduce the risk of this occurring, three separate locations have been identified as having a high likelihood of planned burn smoke exposure during the study period, with the full study being rolled out in two of these three locations. A limitation of this study is exposure misclassification as outdoor measurements will be conducted as a measure for personal exposures. However this misclassification will be reduced as participants are only eligible if they live in close proximity to the monitors.

## Background

Fire has been integral to Australian landscapes for millennia and vegetation across the continent is highly fire adapted and often fire promoting, as some species require severe fires for reproduction. Smoke is therefore a characteristic feature of Australian biophysical environment and intermittent exposure to fire smoke is inevitable for the majority of Australians [1, 2]. Major urban areas, including population centres far from areas of native vegetation, are routinely affected by severe air pollution episodes from landscape fires as smoke can be transported in the atmosphere for long distances [3, 4].

Current evidence suggests that public health harm from bushfires, is likely to be minimised by proactive management through planned burning, rather than relying on reactive management of severe bushfires as they arise [5, 6]. However planned burning to reduce fuel loads, is an important source of exposure to poor air quality [7]. There is strong evidence that extreme pollution from severe, infrequent forest fires contributes to mortality, hospital admissions, and emergency attendances especially for respiratory conditions [8, 9] and some cardiovascular illnesses [10, 11]. However, it is not known if similar risks can be expected at the lower concentrations of smoke derived particulate matter (PM) generally associated with planned burns. The populations affected are usually smaller rural communities that do not lend themselves to large scale population based epidemiological studies because of absent air quality monitoring data, or because relative small populations mean that outcomes such as hospital admissions and deaths are infrequent and not useful for the study of broad episodes of smoke pollution. Evaluation of individual clinical impacts or changes in biomarkers in association with exposure to smoke from planned burns is a practical approach to evaluating potential health impacts of landscape fire smoke in small populations.

Individual clinical and toxicological impacts of urban background PM has been more widely studied than landscape fire derived PM. It is now generally accepted that changes in respiratory function in response to PM occurs through the promotion of inflammation and other pathways including increasing oxidative stress [12]. Impacts on cardiovascular health are thought to be mediated via the promotion of systemic inflammation pro-oxidation, coagulation and changes in heart rhythms from interaction with particles [13].

Most bushfire smoke research to date most has been population based epidemiological studies examining routinely collected hospital or mortality data. [14–17]. However there is an emerging literature examining individual clinical and subclinical endpoints [18]. Health outcomes relevant at an individual level include cardiorespiratory symptoms, medication and health service usage, lung function, airway inflammation, blood pressure, heart rate variability, endothelial function and inflammatory markers in blood. These have been previously investigated in studies of urban air pollution [19–21], but not yet widely applied to bushfires or at all to planned burns.

Studies specific to landscape vegetation fires are uncommon but findings have generally been consistent with the above findings for PM from other, (non-fire) sources. During a series of clinical studies, brief exposure to biomass smoke has been associated with short-term changes in inflammatory cells and other markers [22, 23]. Through in-vivo animal studies, the wildfire-derived PM exposure compared to controls have demonstrated lower counts of lung macrophages, but higher levels of inflammatory cytokines [24, 25]. Finally with in-vitro studies, rat alveolar macrophages exposed to PM_2.5_ from prescribed fires showed increased inflammation compared to controls. Human bronchial epithelial cells exposed to wildfire-derived PM_2.5_ compared to cells exposed to ambient PM also showed increased inflammatory markers [12, 26].

This study is designed to evaluate the relationship between exposure to smoke from planned burns and effects on individual level respiratory and cardiovascular health outcomes.

## Methods/Design

### Aim and Design

The aim is to assess health impacts of smoke, as measured by PM_2.5_ from planned burns in rural communities.

This is a prospective cohort study that is conducted during a planned burning season, the planned burn season occurs in late spring/early summer and late summer/early autumn in the southern hemisphere. Approximately 50 participants undergo three rounds of health assessments over a three to four month period: before, during and after the smoke exposure from planned burning. To ensure we have relatively “clean air” we will need two weeks of no smoke exposure prior to the “before” phase of data collection, this also means that no bushfires or smoke events should take place in or near the collection sites. In addition, the participants keep a diary of respiratory symptoms and lung function for about six weeks. PM_2.5_ exposure data is measured during the planned burning season at a central site in each of the study locations. Every participant will receive standardised individual training, and supervised standard measurements will be collected at the “before” assessment.

### Hypothesis

Our primary hypothesis is that elevation in the levels of PM_2.5_ from planned burning will lead to a decrease in cardiorespiratory health, and increases in inflammatory mediators.

### Setting

The study sites are three towns in the state of Victoria, Australia. We identified three areas where the before measurements are to be conducted. The three study sites have already been identified in discussion with the Victorian Department of Environment, Land, Water and Planning (DELWP). The criteria to determine these three sites are as follows:

- High likelihood of significant smoke exposure from planned burns
- Large enough population to enable recruitment of sufficient participants
- Within three hours drive of Melbourne (due to the short notice we would be unable to fly to the relevant locations)

The three sites chosen to conduct the study in are:

1. Warburton, a town of approximately 2,200 people on the Yarra River within the Little Yarra Valley. Warburton is 72 kilometres from Melbourne.
2. Traralgon, a large regional centre of approximately 24,000 people in the Latrobe Valley region of Victoria. It is approximately 160 kilometres from Melbourne.
3. Maffra and Heyfield are two distinct townships in the Shire of Wellington, Victoria. Heyfield is 206 kilometres from Melbourne and has a population of approximately 2,100 people. Maffra is 220 kilometres from Melbourne with a population of approximately 4,200 people. Due to their relative proximity and similar smoke exposures due to geography, the results from Maffra and Heyfield will be collated as one site.

Most of the sites are visited over several days to collect the health data.

Planned burning operations are unpredictable in timing as they depend on climatic conditions and dryness of the ‘fuel’ (i.e. forest). It is even possible that due to unfavourable conditions no burning takes place in a certain region in a particular season. To minimise the risk of no exposure to smoke in our study, three locations are indicated as suitable, with the full study rolled out in these three locations, depending on smoke exposure.

### Study population

The aim is to study 50 adults, who live in one of the three identified study sites, and are over 18 years of age. As older age is a risk factor for adverse health outcomes from air pollution, we aim to have half the participants aged over 65 years. There are no exclusion criteria based on current health or medical conditions. The only exclusion criterion for participation is, the participant must be aged 18 years and above. Informed consent will be obtained in writing from each participant.

We will collect information on pre-existing diseases and other existing conditions, e.g. we have detailed questions about asthma, COPD, other respiratory diseases. We ask specifically about inflammatory diseases, whether they have high blood pressure and heart conditions (heart failure, arrhythmia etc.) in the questionnaire and these will be included as covariates in analysis.

Whilst the study will require a minimum of 50 participants from across the three sites for statistical power, it is envisaged that the study will attempt to recruit the same number of participants from each site for the study. It is not necessary for the 50 participants to be evenly distributed across all three collection sites. A co-variate will be added in the analysis for location.

There are consistent associations in older persons between bushfire particulate and respiratory hospital admissions [8–10], beyond the underlying effect of exposure to particulates from urban sources. The strongest association with bushfire particulate was for COPD and other respiratory conditions [27].

### Recruitment

Prior to recruitment the study is advertised in the mainstream media, local media, and on social media, followed by recruitment by a professional recruitment/research agency that does random digit calling within the study area. In addition a Facebook page is set up and a free call 1800 number assigned to the study. As an added incentive participants are offered the chance to go into a draw that rewards them with a voucher after each round of measurements.

By using a market research company to target the three site locations to identify potential participants we anticipate a greater number of people will agree to participate in the study. Through cold-calling, people who live in the catchment areas of the study are called on their home phone numbers and asked to participate in the study. A brief outline of the study is explained to them.

In contacting participants we will need to conduct follow-up telephone calls to each person as the dates for the planned burns will be weather dependent. Notice of definite planned burns will be relayed to the study team a few days before the event. The study team will then contact all the participants informing them that the study team will be in the town to carry out data collection on the said days and book a set time for the participant to attend the study.

### Exposure measurements

At a central location in each of the study areas an E-sampler Aerosol Monitor (MetOne Instruments Inc. Grants Pass OR), is set up. The E-sampler measures concentrations of PM_2.5_ (particles with an aerodynamic diameter of less than 2.5 μm). It provide continuous real-time measurements by light –scattering which assists in evaluating peak concentrations, and it also collects gravimetric measurements on filters, which are used to determine a calibration factor for the E-sampler. The weekly gravimetric samples are collected on pre-weighed 47 mm Fluoropore filters, which are also used for analysis of levoglucosan, a biomass burning marker. Participants also keep a daily diary which indicates whether they spent the majority of the day in or outside the study area. The samplers will be deployed for the entire period of the study.

The samplers will be connected to a mains power outlet, and located in an area that is secure and tamper proof. The samplers will be in a central location relative to most participants. In the situation of fire smoke an entire rural community is usually affected by a smoke plume and within each individual community exposure from this source has greater homogeneity of distribution than distributed point sources such as wood heaters.

Additionally we will sample indoor and outdoor Particulate Matter (PM) concentrations at (six) selected individual houses located throughout each study site to evaluate the representativeness of the central monitor and the relationship between indoor and outdoor concentrations of PM. The indoor/outdoor measurements will be spread across the data collection area. One of the requirements is that it will be centrally located in the study area away from any point sources so it would be representative of concentrations in the study area.

The QA/QC program will be similar to that used in previous studies [28, 29]. Gravimetric mass and analytical measurements will be conducted at the NATA- accredited laboratories at CSIRO Oceans and Atmosphere laboratory. Levoglucosan will be determined by high-performance anion-exchange chromatography with pulsed amperometric detection (HPAEC-PAD). Levoglucosan will be separated from other anhydrous sugars such as galactosan and mannosan with a 4 mm × 250 mm CarboPac PA10 analytical column protected by a 4 mm × 50 mm CarboPac PA10 guard column. The development of the HPAEC-PAD technique is based on a previous study to measure levoglucosan in PM_2.5_ from biomass combustion [30]. NATA-accredited gravimetric mass measurements on the pre-exposed and exposed filters will be made using a Mettler UMT2 ultra-microbalance with a specialty filter pan in a temperature and humidity controlled environment.

### Health outcome measurements

Participants visit a central location in each study area where health measurements are performed on three occasions, before, during and after exposure to smoke from planned burns. The data will be collected at three sites that are accessible to the public and located in a venue within or immediate to the town centre.

In addition daily health outcome data is collected and starts on the first (‘before’) visit. The “before” measurements will occur approximately two weeks before the planned burns occur. The study will need to take into consideration the long time frame if we decide to collect data months in advance so we have decided to collect a few weeks prior to the scheduled planned burns in the hope that they will occur as planned. The same staff will collect data from all three sites in the study.

The following data will be collected:

(I) Initial Questionnaire: On the first visit includes questions on age, general health, occupation and period of time living in the study area.
(II) Daily Diary and self-measured lung function for 6–12 weeks
  The participants record in their daily diary information on daily cardiorespiratory symptoms, medication use and health service usage (i.e. GP or hospital attendances) and whether they take measures to reduce their exposure to smoke (i.e. stayed inside as much as possible, reduced outdoor activity, closed windows and doors or wore a mask). The daily diary will ask participants to record inside and outside movement over the course of the study. This will be matched to smoke events over the course of the study.
  Lung function testing: Every morning and evening the participants measure their lung function using a portable electronic PiKo-6 (nSpire Health, Longmont CO) meter. PiKo-6 displays and stores the test results including the ratio of FEV_1_/FEV_6_. Self-measured lung function is less accurate than supervised measurement in a laboratory. However any measurement error is likely to be non-differential meaning that effect estimates would be biased towards the null.
(III) Clinical testing on three occasions (before, during and after smoke exposure)
  Lung inflammation test using an Aerocrine (Aerocrine AB, Solna, Sweden) NiOx Unit which measures exhaled nitric oxide. Exhaled nitric oxide (eNO) is a simple, non-invasive and reproducible method to measure airway inflammation.
  Blood pressure test using an Omron (Omron Global, Kyoto, Japan) Premium Automatic Blood Pressure Monitor HEM-7211
  Heart rate variability and markers of cardiac ischaemia using 24 hour electrocardiography (Holter monitor). This is done on selected participants as we only have 10 monitors available, and we test more than 10 participants per day. Participation in this part of the study will be based on people being willing to wear the Holter monitor for a 24 hour period.
  Endothelial function measured by (i) Finger plethysmography and (ii) flow mediated dilatation of the brachial artery. The finger plethysmography endothelial function measurement is measured via peripheral artery tonometry using the portable EndoPAT 2000 instrument (Itamar Medical Ltd, Cesari, Israel), which calculates the Reactive hyperaemia index. The flow-mediated dilatation (FMD) test is an ultrasound method to assess the ability of the arteries to respond with endothelial NO release during hyperaemia (flow mediated) after a 5 minute occlusion of the brachial artery with a blood pressure cuff. The endothelia measurements will take approximate 30 minutes.
  Blood tests, for blood markers of inflammation and coagulation. Venous blood (around 15 ml) is drawn by experienced field staff. Blood is then centrifuged and the resulting plasma and serum tested for: High sensitivity C-reactive protein, Fibrinogen, and the Full Blood Examination will be performed on whole blood (including haemoglobin, platelet count, White Cell Count). Study researchers/field staff including qualified nurses with training and prior experience in collecting blood samples are employed for the purposes of the study. Additionally appropriate training and supervision is provided to the researchers/field staff who are assisting in conducting other health measurements for the study.

Blood collection will be conducted by either a phlebotomist or registered nurse and stored within the guidelines as recommended by the Australian National Blood Authority in regard to receipt, storage, collection and transportation of blood. The blood will be stored in temperature controlled refrigeration unit specifically used for blood collection and storage. Transport of the blood will be conducted by a specialised courier service that will transport the specimens to a pathology laboratory for analysis. Analysis will take place at the NATA accredited Alfred Hospital Pathology Service laboratories by trained and qualified haematologists or technicians. The blood samples collected will be stored for future analysis by the researchers should funding be made available for subsequent studies.

The study will provide participants with information on any abnormal readings for them to follow-up with their local health provider.

The researchers are first-aid trained and can deal with issues such as breathlessness, feeling light headed, fainting or bruising. There is a review after each health measurement collection to ensure correct data collection procedures. Researchers are made aware of confidentiality agreements. In the case of any adverse/unforeseen events in relation to the collection, use or disclosure of information, the Chief Investigator will inform the Monash University Human Research Ethics Committee (MUHREC) as soon as possible.

### Data management

Data will be retained for at least five years. Storage of data adheres to the University regulations and kept on University premises in a locked cupboard/filing cabinet and password protected database on the University computer network. Only the researchers named in this project will have access to this data. The study results will report aggregated data. No individual’s name will be associated with any published or unpublished report from this study.

### Ethics approval

This research study has been approved by the Monash University Human Research Ethics Committee CF12/3097-2012001570 and the Human Research Ethics Committee of the University of Tasmania, reference number H0013022.

### Statistical analysis

Mixed effect models, adjusting for confounders, will be used for analysis. Exposure to PM_2.5_ is the independent variable and the health outcomes are the dependent variables. For each health outcome a regression coefficient and 95 % confidence interval is calculated for an interquartile range increase in PM_2.5_ (This increase is a realistic increase and will be based on the difference between no smoke exposure and smoke exposure in the study locations). Data is analysed using Stata and R statistical software package.

### Sample size

The sample size for this study (50 subjects) measured on three occasions, is sufficient to find significant effects. Studies using the same end-points have found significant effects using lesser number of participants and observations.

For example two studies related to traffic exposures vs no traffic exposure found significant effects on exhaled nitric oxide [19, 31]. The first study measured 36 participants on three occasions and the second measured 60 participants on two occasions and both found significant associations with eNO. Our estimated sample population of 50 participants measured three times, together with the high likelihood that the PM_2.5_ concentrations in the two studies are lower than the concentrations that will occur in the present study, will be sufficient to find an effect if there is one.

Another example is an intervention study from Canada using HEPA filters indoor, which investigated the association between wood smoke exposure and RHI (marker of endothelial function) and C-reactive protein (CRP - marker of inflammation in the blood) in 45 participants on two occasions. They found significant associations for both RHI and CRP whereas concentrations are expected to be an order of magnitude higher in our study [32].

Previous studies have suggested that increased exposure to PM_2.5_ is associated with increased levels of CRP [33]. Based on the largest study conducted in a general population we estimated the mean population CRP level will be approximately 2.7 mg/L with a standard deviation of 4.9 mg/L [34]. Previous effect sizes have shown varied results [35]. A previous study indicated that a 99.1 % increase in CRP was observed per 34.5 ug/m^3^ increase in PM_2.5_ [10]. Although this study was conducted in younger patients, which tend to have a lower baseline CRP level (0.8 mg/L in this study), the exposure difference in our study is anticipated to be larger; approximately 60 μg/m^3^ difference in PM_2.5_ levels between the unexposed and exposed periods. A US study in older persons (mean CRP = 2.4, SD = 3.2), suggested a change of 0.75 mg/L per 7.0 ug/m^3^ of PM_2.5_ [13]. Thus for an increase of 60 μg/m^3^ in PM_2.5_ we’d expect to see an increase of 6.43 mg/L in CRP [(60/7)*0.75]. Using a population mean of 2.7, a conservative expected increase of 2.7 with a population standard deviation of 4.9, the estimated sample size for two-sample comparison of mean CRP levels can be calculated as 52. As CRP is our primary outcome in our study we have powered this study to be able to see a signal on this biomarker. However we will also be investigating the associations between PM_2.5_ and the other biomarkers, like fibrinogen.

## Discussion

A strength of this study is that it collects individual level health data and not population level data. This allows investigation of subclinical health outcomes which has not been done to date in relation to planned burn smoke exposure in the community[36]. Another strength is the large difference in particulate matter concentration during smoke exposure compared to no smoke exposure. Most studies have investigated urban air pollution or traffic air pollution and these differences are about an order of magnitude lower than what is experienced during smoke from planned burns in the local communities.

One of the key challenges involved in a study such as this is the process whereby the study team is informed of the dates of the planned burns by the relevant authorities and the short time frame that will be required to contact potential participants. The aim is to perform the ‘before’ assessment within 2 weeks of the planned burns occurring. The assessment after the burns will be done once there has been no smoke in the area for 2 weeks. That should be enough time for the health measures to return to baseline and give an indication of possible long term effects of the smoke if measures have not returned to baseline levels at this stage. As the study is susceptible to changes in weather, it is proposed that data collection will occur either during the spring or autumn to reduce the possibility of data collection being compromised by bushfires during the warmer summer months, or heavy rains during the winter months [37]. The authorities conducting the planned burns require that there is no rainfall several days prior to a planned burn, and that no rain falls during the planned burn. This is to maximize the opportunity to burn off the undergrowth that feed bushfires. This can lead to the study being delayed or postponed if weather conditions are not favorable for the planned burns. As such the study will need to keep participants informed of potential follow-up dates with the possibility of sudden cancellations. This may also lead to potential participants not being available on the date collection days are due to take place because of other commitments.

A limitation of this study is exposure misclassification. Air pollution measurements are conducted at a central location at each study location. However as we do not have personal exposures during the study this may lead to misclassification as the outdoor concentration is not necessarily the same as the personal exposure of an individual participant. However it is common practice to use outdoor exposure estimates as a proxy for personal exposure in air pollution epidemiology. We collect daily information on the amount of time the participant resides in the study area and this is used to reduce exposure misclassification.
